# Free Flap Reconstruction for Gastrocnemius Muscle Necrosis to Avoid Above-Knee Amputation

**DOI:** 10.70352/scrj.cr.25-0498

**Published:** 2026-03-04

**Authors:** Hikaru Watanabe, Suzuna Ishimoto, Maiko Inada, Miki Nakanishi, Yasuhiro Sakata, Satsuki Tachibana, Shinichi Asamura

**Affiliations:** Department of Plastic and Reconstructive Surgery, Graduate School of Medicine, Wakayama Medical University, Wakayama, Wakayama, Japan

**Keywords:** below-knee amputation, free tissue flaps, free latissimus dorsi flap, gastrocnemius muscle necrosis, stump reconstruction, above-knee amputation, functional outcome

## Abstract

**INTRODUCTION:**

Below-knee amputation is functionally superior to above-knee amputation, and preservation of the knee joint is essential for optimal mobility. In below-knee amputation, muscular coverage of the tibial stump and proper shaping for prosthesis fitting are key factors in functional recovery. If primary muscle coverage is not feasible, free flap reconstruction may be required to achieve these goals.

**CASE PRESENTATION:**

A 23-year-old man presented with gait disturbance due to complete peroneal nerve palsy, tibial nerve palsy, and disuse atrophy of the gastrocnemius muscle following a traffic accident at the age of 19 years. MRI suggested necrosis of the gastrocnemius muscle, which made primary coverage of the tibial stump unfeasible. To avoid the poor functional prognosis associated with above-knee amputation and to preserve the knee joint, we preoperatively planned and successfully performed a below-knee amputation with free latissimus dorsi musculocutaneous flap reconstruction. The patient achieved independent ambulation and was able to run with a prosthesis 10 months postoperatively. Three years after surgery, he remains free of complications and reports good functional outcomes.

**CONCLUSIONS:**

This case highlights that below-knee amputation with free flap reconstruction should be considered in functionally independent patients for the preservation of activities of daily living (ADL) and maintaining long-term QOL.

## Abbreviations

AKA
: above-knee amputation
BKA
: below-knee amputation
LD
: free latissimus dorsi musculocutaneous flap transfer

## INTRODUCTION

In below-knee amputation (BKA), surgery aims to facilitate adequate bone stump coverage by the use of muscle tissue and creation of a stump for which a prosthesis can be used. If the muscle is insufficient to cover it or otherwise cannot be used, then above-knee amputation (AKA) is often considered. Nonetheless, the preservation of the knee joint, irrespective of whether AKA or BKA, plays a critical role in determining functional outcomes. We report a case in which a free latissimus dorsi musculocutaneous flap transfer (LD) was used to reconstruct a below-knee stump in a patient with necrosis of the gastrocnemius muscle. This approach allowed for knee joint preservation and led to a favorable postoperative course.

## CASE PRESENTATION

**Fig. 1:**
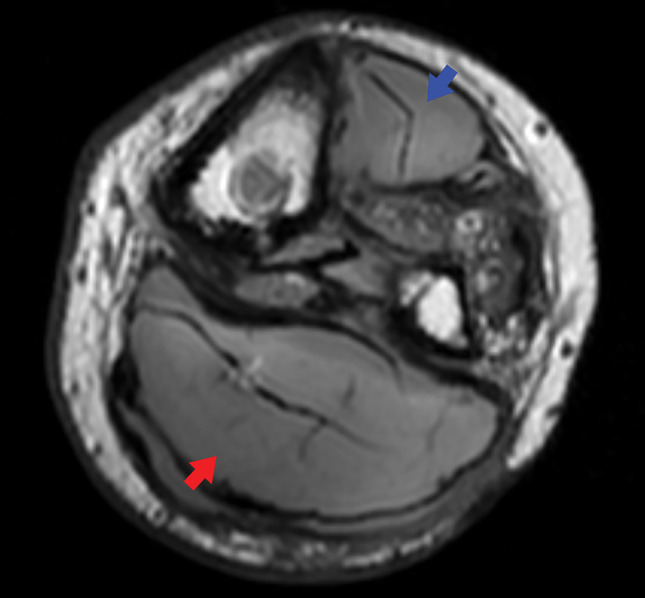
Axial MRI of the left lower leg at the initial presentation. Necrotic changes are observed in the tibialis anterior muscle (blue arrowhead) and the triceps surae (gastrocnemius–soleus) muscle (red arrowhead).

The patient was a 23-year-old man who had sustained multiple traumatic injuries at the age of 19 years following a motor vehicle accident. His injuries included soft tissue injury with skin defects in the left thigh and multiple open fractures of the extremities (left tibia and fibula, right ulna and radius) as well as extensive visceral trauma. There was no vascular injury to the popliteal artery or popliteal vein. Fracture and prolonged bed rest resulted in complete peroneal nerve palsy that caused foot drop, tibial nerve palsy, and disuse atrophy of the gastrocnemius muscle. Multiple ulcerations developed due to sensory impairment. Despite undergoing multiple foot drop surgeries, the patient was unable to walk. After 4 years of ongoing treatment at another hospital without achieving the ability to walk, lower limb amputation with the goal of prosthetic walking was considered, and the patient was referred to our department. On initial presentation, the left lower leg exhibited multiple scars from recurrent ulceration, foot drop, and sensory deficits in the distributions of both the common peroneal and tibial nerves. MRI revealed necrosis of the tibialis anterior and gastrocnemius muscles (**Fig. 1**). During BKA, coverage of the tibial stump was deemed unfeasible, so LD was utilized to achieve adequate stump coverage.

### Surgical procedures

**Fig. 2:**
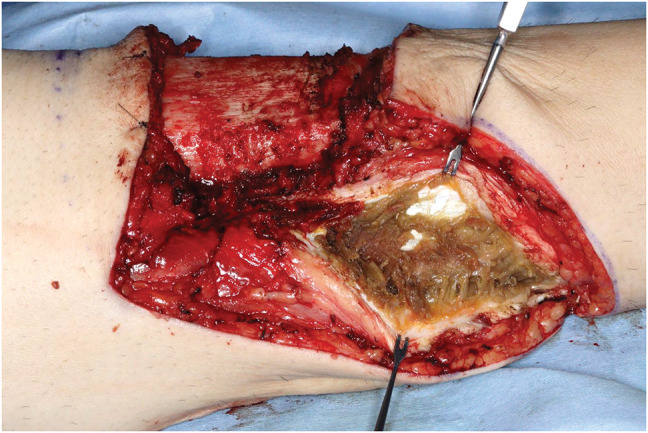
Intraoperative view from the medial side of the left lower limb. Necrosis of the gastrocnemius muscle located dorsal to the tibial stump is observed.

The skin incision was designed in a fish-mouth configuration, and the tibial osteotomy was made 12 cm distal to the knee joint line and performed following the Burgess technique.^1)^ Intraoperatively, the gastrocnemius muscle was found to be completely necrotic from its origin (**Fig. 2**), rendering it unsuitable for coverage of the tibial stump. Following thorough debridement, we harvested the LD from the right side with a skin paddle measuring 6.5 × 27 cm, and we used it for the stump coverage, as we had preoperatively planned. Microvascular anastomosis was performed end-to-end between the thoracodorsal artery and vein and the popliteal artery and vein. The thoracodorsal nerve and intercostal nerves were coapted end-to-end to the sciatic nerve. The latissimus dorsi muscle was anchored to the tibial stump.

### Postoperative course

**Fig. 3:**
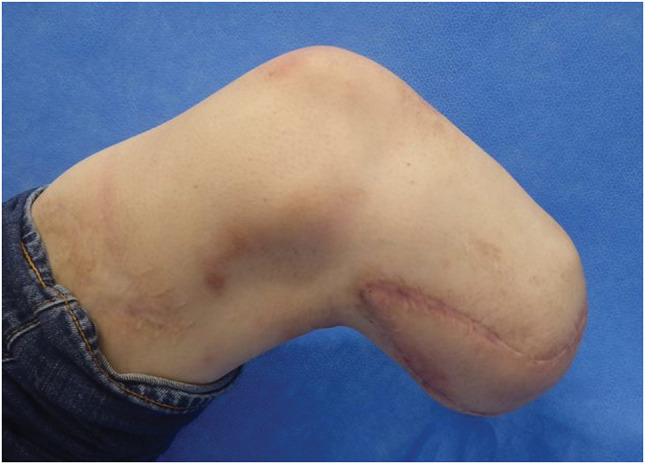
Eighteen months after surgery.

**Fig. 4:**
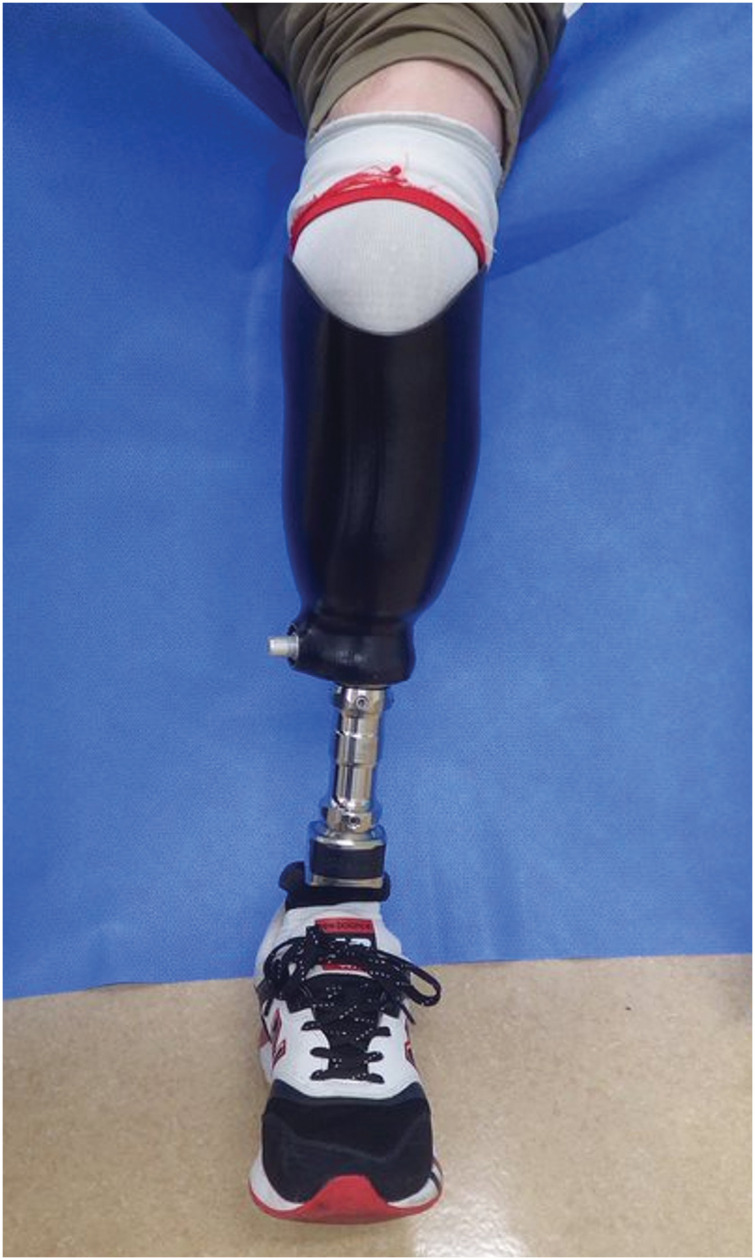
With prosthesis attached.

Postoperatively, the flap remained viable without any signs of wound dehiscence or partial skin necrosis, and complete flap integration was achieved. Rehabilitation with the use of a prosthetic limb and crutches was initiated 3 weeks after surgery, and the patient was discharged home approximately 1 month postoperatively. By 3 months, he was able to ambulate with a cane; by 6 months, he could walk independently without assistive devices; and by 10 months, he was capable of running. At the 3-year follow-up, the patient remained free of complications and reported good functional outcomes. Neurologically, the Semmes–Weinstein monofilament test yielded a score of 6.65, indicating a loss of protective sensation. Although superficial sensation was absent, deep pressure sensation was preserved (**Figs. 3** and **4**). The patient provided informed consent to the publication of the details of his case and the accompanying images.

## DISCUSSION

BKA and AKA differ a great deal in terms of functional outcomes. BKA, for example, is associated with lower energy expenditure and higher rates of ambulation with prosthetic use than AKA.^2)^ Reported prosthetic walking rates are 34%–40% for BKA and 9%–20% for AKA.^3,4)^ Regarding walking speed, 1 group reported approximately 1.2 m/s for patients with AKA and approximately 1.6 m/s for patients with BKA. Energy expenditure during walking was said to increase by 30%–60% in patients with AKA and 0%–15% in patients with BKA compared to able-bodied individuals.^2)^ Preserving the knee joint via BKA is thus thought to be important for maximizing activities of daily living (ADL).

In BKA, muscle tissue is used to cover the residual bone, and it is important to have proper stump formation to enable the use of a prosthesis. In our patient’s case, however, the residual bone could not be covered by the gastrocnemius muscle, so we preoperatively planned reconstruction using a free flap. For stump reconstruction, thick tissue capable of withstanding prostheses pressure is ideal.^5)^

The LD is larger than other muscle flaps and has less subcutaneous fat, making it suitable for covering the lower leg stump, which is subject to high pressure.^5)^ Additionally, performing surgery in the lateral decubitus position eliminates the need for intraoperative repositioning, it allows for attempts at sensory reconstruction via nerve suturing, and it minimizes adverse effects from skin flap harvesting during postoperative rehabilitation—all of which are thought to be advantageous in lower leg amputations. Although a filet flap might have been ideal because it avoids sacrificing healthy tissue,^6)^ it was contraindicated in this case due to recurrent heel ulceration. The anterolateral thigh flap has a reported association with limited high-performance mobility, such as running.^5,7)^ We suggest the rectus abdominis muscle flap is unsuitable for stump formation because it requires positional changes during surgery, has a high fat content that can become bulky, and may require additional surgery for fat reduction to match the prosthesis. We therefore opted to use the LD in this case. Additionally, considering the potential contact between the flap harvest site and crutches, as well as the significant load on the amputated limb side, we opted to harvest from the contralateral side.^5)^

Atrophy and sensory deficits at the amputation site are challenges associated with free flaps. These can pose a significant risk for the development of difficult-to-heal ulcers with prosthesis use, but sensory reconstruction may contribute to improvement.^8,9)^ However, there are also reports of the sensory flap gaining sensory recovery comparatively rapidly, which may aid in preventing ulcerations,^5)^ but several studies have noted a lack of statistical difference regarding skin breakdown in long-term follow-up periods, irrespective of whether they performed a sensory flap.^5,10,11)^ Muscle was reportedly sensitive to pressure and patients felt deep pressure at the distal stump, which was sufficient to prevent skin breakdown.^5,12)^ In our patient’s case, superficial sensation was not restored, but no ulcers or atrophy were observed at 3 years postoperatively. Preservation of deep pressure sensation might have contributed to this outcome, but evaluation is difficult in a single case study, and comparative analysis with other cases is necessary in the future.

After exploring the potential advantages and disadvantages of both AKA and BKA, we performed BKA in this case. The loss of a limb can be a significant psychological burden for patients. Given the young age of our patient, the potential psychological burden and the associated functional prognosis, we decided that it was important to preserve as much lower limb length as possible. Although long-term follow-up is necessary, this case supports the usefulness of avoiding AKA whenever possible and preserving the knee joint in functionally independent patients to achieve favorable outcomes in QOL and ADL. It also suggests that using a free flap in lower leg amputation may be a useful method for avoiding AKA.

## CONCLUSIONS

This case demonstrates that in functionally independent patients, avoiding femoral amputation whenever possible and preserving the knee joint during lower limb amputation should be considered not only for maintaining ADL but also for preserving long-term QOL. Furthermore, it suggests some benefits of using free flaps for lower leg amputation.

## DECLARATIONS

### Availability of data and materials

Data will be made available on reasonable request.
